# CXCR2 deficient mice display macrophage-dependent exaggerated acute inflammatory responses

**DOI:** 10.1038/srep42681

**Published:** 2017-02-16

**Authors:** Douglas P. Dyer, Kenneth Pallas, Laura Medina Ruiz, Fabian Schuette, Gillian J. Wilson, Gerard J. Graham

**Affiliations:** 1 Chemokine Research Group, Institute of Infection, Immunity and Inflammation, College of Medical, Veterinary and Life Sciences, University of Glasgow, 120 University Place, Glasgow G12 8TA, UK

## Abstract

CXCR2 is an essential regulator of neutrophil recruitment to inflamed and damaged sites and plays prominent roles in inflammatory pathologies and cancer. It has therefore been highlighted as an important therapeutic target. However the success of the therapeutic targeting of CXCR2 is threatened by our relative lack of knowledge of its precise *in vivo* mode of action. Here we demonstrate that CXCR2-deficient mice display a counterintuitive transient exaggerated inflammatory response to cutaneous and peritoneal inflammatory stimuli. In both situations, this is associated with reduced expression of cytokines associated with the resolution of the inflammatory response and an increase in macrophage accumulation at inflamed sites. Analysis using neutrophil depletion strategies indicates that this is a consequence of impaired recruitment of a non-neutrophilic CXCR2 positive leukocyte population. We suggest that these cells may be myeloid derived suppressor cells. Our data therefore reveal novel and previously unanticipated roles for CXCR2 in the orchestration of the inflammatory response.

The chemokine family is comprised of approximately 45 structurally related proteins that, along with their cognate G-protein coupled receptors (GPCRs) expressed on circulating cells^1^, mediate the *in vivo* migration and recruitment of leukocytes^2^,^3^. Broadly speaking chemokines, and their receptors, can be defined as being either inflammatory or homeostatic according to the contexts in which they function^4^,^5^. Inflammatory chemokines play specific roles in choreographing leukocyte migration in response to inflammatory or infectious stimuli, however the precise functional roles played by individual chemokines and their receptors in this complex process remain poorly defined. Not surprisingly, inflammatory chemokines and their receptors are essential contributors to a range of immune and inflammatory pathologies. They also play fundamental roles in tumourigenesis^6^,^7^,^8^,^9^. Overall therefore, the inflammatory chemokine/receptor system represents an important therapeutic target although attempts at developing therapeutically useful antagonists of chemokine receptor have so far met with extremely limited success^10^,^11^. There are many potential reasons for this one of which is our, currently, poorly developed understanding of the complexity of the inflammatory chemokine/receptor system and its role in regulating inflammation. We contend that a clearer understanding of this system is required before chemokine receptor antagonists can be used effectively as therapeutic agents.

Despite the general lack of success, one promising target currently being pursued is the neutrophil-expressed chemokine receptor CXCR2 which, alongside its ligands CXCL1–3 and CXCL5–8, regulates neutrophil recruitment in a number of pathological contexts^12^,^13^. Thus it is a viable therapeutic target in, for example, COPD and other neutrophilic inflammatory pathologies. In addition, recent experimental evidence has highlighted an important role for CXCR2 in cancer formation and maintenance^8^. Specifically, targeting of CXCR2 has resulted in suppression of inflammatory skin and intestinal tumorogenesis^14^, reduced spontaneous adenocarcinoma formation in the intestine^14^, decreased acute myeloid leukemia^15^, inflammatory bowel disease^16^, nasopharyngeal carcionoma^17^ and rhabdomyosarcoma (in combination with anti-PD1)^18^. Accordingly, small molecule antagonists of CXCR2 are being actively tested for potential anti-cancer effects *in vivo*.

In this study we looked, in detail, at the impact of CXCR2 deletion on the kinetics of the inflammatory response. We now report a counterintuitive transient exaggeration of inflammatory response in CXCR2-deficient mice in models of both skin and peritoneal inflammation. This phenotype was associated with increased accumulation of macrophages at inflamed sites in CXCR2-deficient mice and was dependent on CXCR2-driven recruitment of a suppressor cell population to the acutely inflamed environment. We hypothesise that these cells may be myeloid derived suppressor cells. In summary, our study reveals a novel role for CXCR2 in limiting the magnitude of the macrophage-dependent inflammatory response.

## Results

### CXCR2-deficient mice display excessive cutaneous inflammatory responses

**Figure 1:**
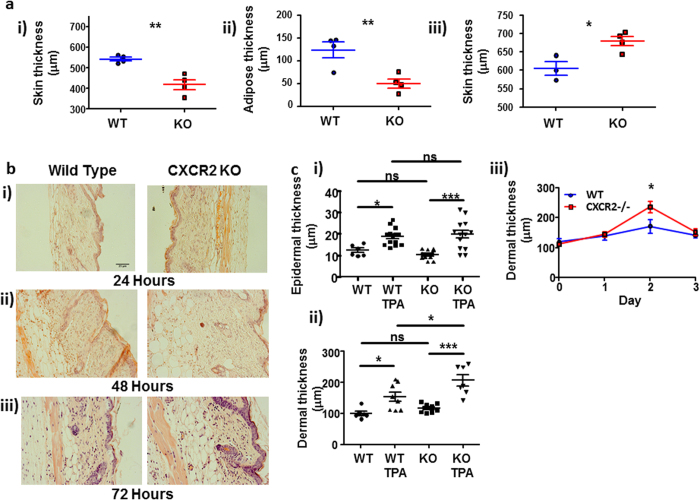
CXCR2-deficient mice display excessive cutaneous inflammatory responses. **(a)** The dorsal skin thickness of **(i)** resting or **(iii)** TPA treated (48 hours) wild type or CXCR2-deficient mice was measured using callipers and the adipose layer thickness **(ii)** of resting skin was digitally measured by microscopy on haematoxylin and eosin stained skin sections. **(b)** Dorsal skin from WT and CXCR2-deficient mice treated with TPA for **(i)** 24, **(ii)** 48 or **(iii)** 72 hours were sectioned and stained with haematoxylin and eosin and imaged using light microscopy. **(c)** Stained sections from skin harvested 48 hours after vehicle or TPA treatment were analysed to measure **(i)** epidermal and **(ii)** dermal thickness. **(iii)** dermal thickness is also shown from sections harvested over the post-TPA application period. **a and ciii:** Data are plotted as the mean (±SEM) of one experiment undertaken with 3–6 individual mice, representative of at least two independent experiments *P < 0.05, **P < 0.01, ***P < 0.001 as determined using Students T test. **Ci and Cii:** Data plotted as the mean (± SEM) of two experiments each undertaken in 4–7 mice *P < 0.05, **P < 0.01, ***P < 0.001 as determined using repeated measures ANOVA analysis with Tukey’s post hoc test.

We initially hypothesised that CXCR2-deficient mice would demonstrate a reduced inflammatory response to the phorbol ester TPA due to the key role that CXCR2 plays in neutrophil migration. To test this we measured total skin thickness (a surrogate marker of inflammation) using calipers. Interestingly, measurement of resting skin thickness showed CXCR2-deficient skin to be significantly thinner than WT skin (Fig. 1ai). This was not due to differences in dermal or epidermal thickness but to a marked reduction in the thickness of the sub-dermal adipose layer (Fig. 1aii). Given that the average difference in the thickness of the adipose layer was 75 μm, and that 2 skin thicknesses were measured using the calipers, this difference in adiposity fully accounts for the resting differences in full skin thickness. Strikingly, and despite these resting differences, our data show that 48 hours after application of TPA to the lower dorsal skin of CXCR2-deficient mice, skin thickness was significantly increased compared to that in WT mice (Fig. 1aiii) indicative of increased inflammation. This was confirmed by haematoxylin and eosin staining. Importantly, this time point is delayed relative to the peak of neutrophil infiltration into the skin in this model (6–24 hrs)^14^. Gross imaging of the stained sections (Fig. 1b) demonstrated that whilst there were no apparent differences in thickness of WT and CXCR2-deficient skin at 24 or 72 hours, CXCR2-deficient skin was significantly thicker, than WT, 48 hours after TPA application (Fig. 1bii). This was quantified by systematically measuring epidermal and dermal thickness at 48 hours post-TPA application (Fig. 1c). These data demonstrated a similar modest TPA-dependent increase in epidermal thickness in both WT and CXCR2-deficient mice (Fig. 1ci) but a significantly greater increase in thickness of the dermal compartment in CXCR2-deficient, compared to WT, mice (Fig. 1cii) that was transient and gone by 72 hours (Fig. 1ciii).

TPA induces hyperproliferation of the epidermis and an influx of proliferating leukocytes. Ki67 staining of skin sections revealed increased epidermal proliferation in both WT and CXCR2-deficient mice at all-time points examined, with strongly increased proliferation of resident dermal cells at 48 hours post-TPA application selectively in the CXCR2-deficient mice (Supplementary Fig. 1a). This was quantified (Supplementary Fig. 1b) demonstrating that the increased numbers of Ki67+ve dermal cells in CXCR2-deficient mice reached significance 48 hours post TPA application. This indicated an exaggerated dermal inflammatory response in the CXCR2-deficient mice which was also reflected in significantly increased size of skin draining lymph nodes at both 48 and 72 hours post TPA application (Supplementary Fig. 1c). Thus CXCR2 deficient mice display exaggerated cutaneous inflammatory responses.

### Increased macrophage numbers are associated with exaggerated cutaneous inflammation in CXCR2-deficient mice

**Figure 2:**
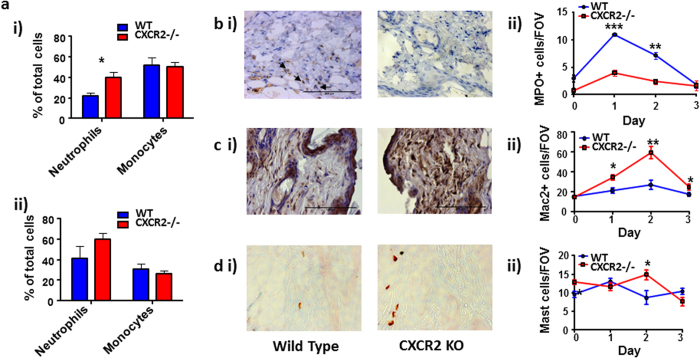
Increased macrophage numbers are associated with exaggerated cutaneous inflammation in CXCR2-deficient mice. **(a**) Cytospin analysis was used to determine the neutrophil and monocyte-like cell content in **(i)** blood or **(ii)** bone marrow of WT and CXCR2-deficient mice. Skin sections from WT or CXCR2-deficient mice treated with TPA (harvested 48 hours after TPA application) were stained and analysed to measure the levels of **(bi)** myeloperoxidase+ve cells, **(ci)** MAC2+ve cells or **(di)** mast cells The kinetics of accumulation of these cell types are shown (0, 24, 48 and 72 hours after TPA application) in **bii, cii** and **dii**. Data are plotted as the mean (±SEM) of one experiment undertaken with 3–6 individual mice, representative of at least two independent experiments *P < 0.05, **P < 0.01 as determined using Students T test.

Notably, and as previously reported^12^, CXCR2-deficient mice display a modest but significant increase in the numbers of circulating neutrophils but no difference in the numbers resident in the bone marrow (Fig. 2ai and ii). No differences in the numbers of either bone marrow, or circulating cells with a monocyte-like morphology were detected in the CXCR2-deficient mice. Given the well-established role of CXCR2 in mediating neutrophil recruitment^19^, we examined neutrophil numbers in WT and CXCR2-deficient mouse skin following TPA application. Myeloperoxidase staining of sectioned skin (shown for 48 hour skin, Fig. 2bi) confirmed that there was significantly reduced neutrophil recruitment 24 hours and 48 hours after TPA application in CXCR2-deficient mice; whilst little, if any, positive staining was present in resting skin or 72 hours after TPA application in WT or CXCR2-deficient mice (Fig. 2bii). Skin was then analysed for expression of MAC2, a macrophage marker^20^ (shown for 48 hours skin, Fig. 2ci). This revealed that MAC2+ve cells are present at significantly higher numbers in the CXCR2-deficient, compared to WT, skin at all time-points measured reaching a peak 48 hours after TPA application. Quantification of macrophage numbers indicated that the strong influx of macrophages was selectively observed in CXCR2-deficient skin whereas little change in staining was seen across these time points in the WT skin (Fig. 2cii). Finally, Astra blue (a mast cell stain^21^) analysis of the same sectioned skin samples revealed modestly increased mast cell numbers at 48 hours after TPA application in CXCR2-deficient mice, compared to WT mice (Fig. 2di). Again little, if any, increase in mast cell presence is observed across the time points analysed in the skin of WT mice (Fig. 2dii) and the higher basal levels of mast cells in resting CXCR2-deficient skins suggest that inflammation, overall, had little effect on mast cell numbers in either strain. Thus the excessive inflammatory phenotype in CXCR2-deficient mice correlates predominantly with the presence of macrophages (MAC2+) and the absence of neutrophils.

### Inflamed CXCR2-deficient skin contains reduced levels of anti-inflammatory, and increased levels of pro-inflammatory, cytokines

**Figure 3:**
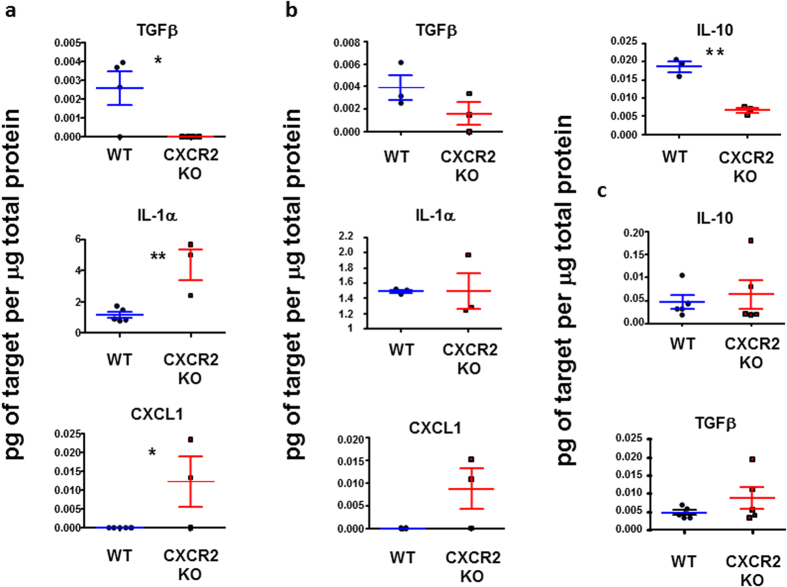
Inflamed CXCR2-deficient skin contains reduced levels of pro-resolution, and increased levels of pro-inflammatory, cytokines. Skin was harvested, and frozen, at either **(a)** 24 hours, **(b)** 48 hours or **(c)** 72 hours following application of TPA to the skin of WT, or CXCR2-deficient, mice. The tissues were then ground in liquid nitrogen, re-suspended in PBS and analysed using a multiplex cytokine and chemokine array. Data are plotted as the mean (±SEM) of one experiment undertaken in 3 mice, representative of two independent experiments. *P < 0.05, **P < 0.01, ***P < 0.001 as determined using Students T test.

We next used multiplexing approaches to measure the concentrations of pro and anti-inflammatory cytokines in the inflamed skins of WT and CXCR2-deficient mice. Skin samples were collected 24, 48 and 72 hours after TPA application and protein extracted by grinding in liquid nitrogen. Data from the multiplexing analysis revealed few significant differences between WT and CXCR2-deficient mouse skin however those that were detected are shown in Fig. 3a (24 hours), b (48 hours) and c (72 hours). These data indicate that TGF-β remained undetectable in skin from CXCR2-deficient mice, compared to WT, at 24 hours and levels remained suppressed at 48 hours. With IL-10, another anti-inflammatory cytokine, levels were not significantly different between WT and CXCR2-deficient mice at 24 hours (data not shown), however at 48 hours post-TPA application there were approximately 5-fold lower levels of IL-10 in CXCR2-deficient mice, compared to WT. 72 hours after TPA application both IL-10 and TGF-β levels were comparable in the wild type, and CXCR2-deficient, mouse skin and higher than at the earlier time points consistent with the ongoing resolution of the inflammatory response. Conversely there was significantly more IL-1α present in the CXCR2 null mouse skin 24 hours post-TPA application although this was not maintained at 48 hours. Furthermore, and in contrast to WT mice, the CXCR2 ligand CXCL1 was readily detectable in the CXCR2-deficient mouse skin at both 24 and 48 hours. This is in agreement with previous findings suggesting that the chemokine ligand of a receptor is often detected at greater levels in its absence due to the lack of scavenging^22^,^23^. Overall, these findings suggest that reduced levels of anti-inflammatory cytokines, and increased levels of pro-inflammatory cytokines, may contribute to the excessive skin inflammation in CXCR2-deficient mice.

### CXCR2-deficient mice display increased peritoneal inflammatory responses

**Figure 4:**
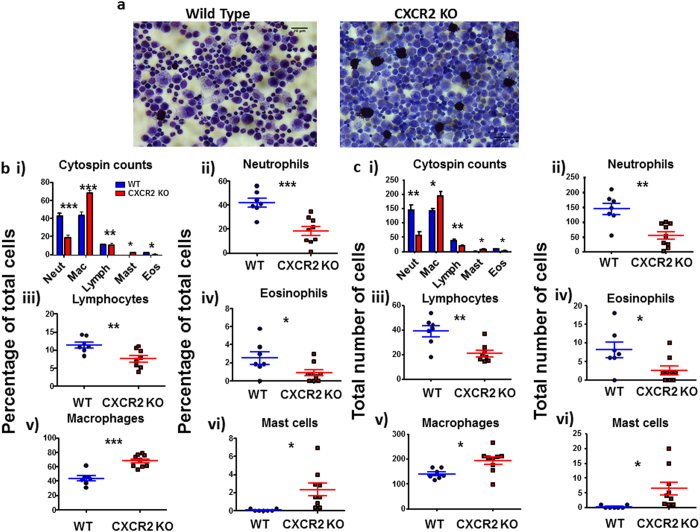
CXCR2-deficient mice display increased peritoneal inflammatory response. Zymosan was intra-peritoneally injected into mice and, 48 hours later, the mice were culled and the peritoneum flushed with PBS. **(a)** Cells were cytocentrifuged onto slides and subjected to differential analysis to identify component cell types (images shown at x40 magnification). Counts are expressed as a percentage of the total cells (**b**) or as the total number of cells per field of view **(c).** Cell counts were taken from at least 5 fields of view per sample and are plotted (for the indicated cell types) as the mean (±SEM) from groups containing at least 7 mice. These plots are representative of two independent experiments. *P < 0.05, **P < 0.01, ***P < 0.001 as determined using Students T test.

In order to examine whether the inflammatory abnormalities in CXCR2-deficient mice were specific for cutaneous inflammation we next analysed the peritoneal inflammatory response to injection of zymosan. This response was measured at 48 hours post zymosan administration, after the peak of neutrophil recruitment in this model^24^, in order to act as a comparison to the observations from the cutaneous inflammatory model. Morphological analysis of the peritoneal lavage following cytocentrifugation (Fig. 4a) revealed a significantly higher density of inflammatory cells in the peritoneal lavage in CXCR2-deficient, compared to WT, mice. Quantitative analysis of cells in these ‘cytospins’ revealed a significant decrease in relative (Fig. 4b), and total (Fig. 4c), neutrophil numbers and, again, a significant increase in macrophage numbers in CXCR2-deficient, compared to WT, mouse peritoneal exudate (Fig. 4bi). More detailed analysis of specific cell morphology revealed, as expected, a significant reduction in the neutrophil infiltrate in the CXCR2-deficient mice (Fig. 4bii) along with a more modest reduction in the numbers of lymphocytes and eosinophils (Fig. 4biii and iv). Again, and in keeping with the data from the skin analysis, there was a highly significant increase in the number of macrophages present in the peritoneal lavage from the CXCR2-deficient mice (Fig. 4bv) which also displayed an enhanced proportion of mast cells (Fig. 4bvi). The same pattern between cells was observed when analysed as a relative percentage (Fig. 4b) and as a total number of cells per field of view (Fig. 4c). These differential counts were confirmed by flow cytometric analysis of the cellular content of the peritoneal lavage (specimen plots are shown in Supplementary Fig. 2a) which revealed an increase in overall cell number, and specifically of CD11b+ cells, in CXCR2-deficient mice (Supplementary Fig. 2b). The flow cytometric analysis also confirmed the reduction of Siglec-F+ eosinophils in the peritoneal lavage in CXCR2-deficient mice.

**Figure 5:**
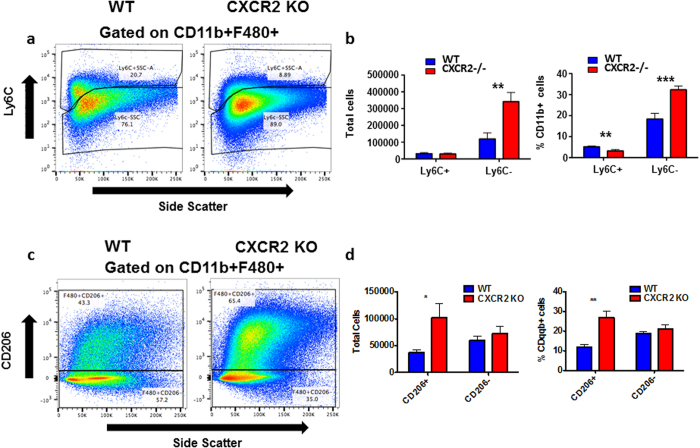
CXCR2 null mice have increased levels of resident macrophages 48 hours after intra-peritoneal administration of zymosan. Zymosan was intra-peritoneally injected into mice, 48 hours later mice were culled and the peritoneum flushed with PBS and cells were then stained with antibodies against the indicated epitopes and analysed using flow cytometry, Cells were gated to select CD11b+F480+cells (as indicated in Supplementary information), these were then assessed for expression of Ly6C (A) and CD206 (C). Quantitative data from the flow cytometric assessments of Ly6C (B) and CD206 (D). Data are plotted as the mean (±SEM) from groups containing at least 7 mice. **P < 0.01, ***P < 0.001 as determined using Students T test.

Further analysis (Fig. 5a) of the recruited excess macrophage population in CXCR2-deficient peritoneum revealed them to be predominantly CD11b+F480+Ly6Clo. These cells were significantly increased in number (Fig. 5b) in CXCR2-deficient inflamed peritoneum. In contrast Ly6C+ cells were relatively under-represented in CXCR2-deficient peritoneum. Interestingly, the CD11b+F480+Ly6Clo cells did not stain positive for CCR2 expression (data not shown) which, together, suggests that they are likely to be derived from non-classical patrolling^25^, tissue repair, monocytes recruited to the inflammatory site. These findings are further strengthened by our data indicating that in the CXCR2-deficient peritoneum there is a significant increase in the number and proportion of CD11b+F480+CD206+macrophages (Fig. 5c and d). These macrophages have been traditionally described as alternatively activated (M2) and further indicate a skew towards this population during the inflammatory response in CXCR2 null mice^26^. Thus CXCR2-deficient mice display an exaggerated inflammatory response to peritoneal administration of zymosan characterised by increased accumulation of macrophages with a ‘patrolling, tissue-repair’ phenotype.

### Analysing the mechanistic basis for the phenotpe

**Figure 6:**
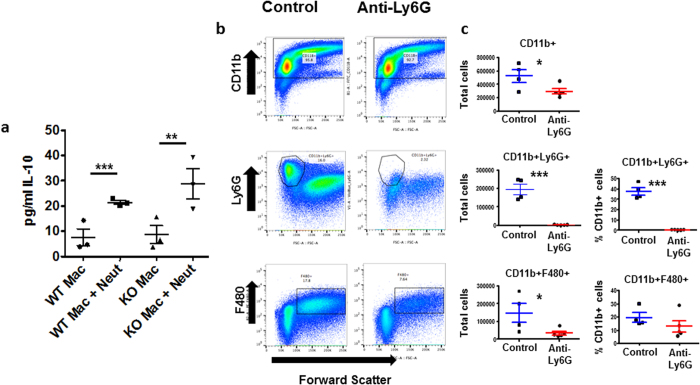
Analysis of neutrophil involvement in the exaggerated inflammatory response. (**A)** Macrophages from WT and CXCR2-deficient mice and WT aged-neutrophils purified from the peritoneum of thioglycollate-injected mice were either incubated alone, or co-incubated for 24 hours. Culture media was analysed with an Elisa for IL-10 protein levels. Plotted as the mean (±SEM) of one experiment undertaken in triplicate. **P < 0.01; ***P < 0.001 as determined using repeated measures ANOVA analysis with Tukey’s post hoc test. (**B)** Either control or anti-Ly6G antibody (500 μg) was injected into the mouse peritoneum (to deplete neutrophils) 24 hours before and after injection of zymosan (1 mg). 48 hours after zymosan injection mice were culled and the peritoneum flushed with PBS and the resulting lavage analysed using flow cytometry against the indicated epitopes. **(C)** Quantitative data from the flow cytometric assessments. Data are plotted as the mean (±SEM) from groups containing at least 4 mice. *P < 0.05, ***P < 0.001 as determined using Students T test.

Previously a number of studies has demonstrated an important role for apoptotic neutrophils in triggering anti-inflammatory responses through interaction with macrophages^27^,^28^. We therefore hypothesised that the exaggerated inflammation seen in CXCR2-deficient mice was a direct consequence of impaired neutrophil recruitment and associated reduction of macrophage-dependent anti-inflammatory cytokine release. Therefore, we directly tested the effects of apoptotic neutrophils on the macrophage ‘resolution’ phenotype. Intraperitoneal injection of thioglycollate was used to obtain neutrophils, which were then aged overnight to induce apoptosis. Inflammatory macrophages were also collected from thioglycolate-stimulated peritoneum of WT and CXCR2-deficient mice. Aged neutrophils, and purified macrophages, were then co-cultured for 24 hours and the cells separated and analysed for expression of the pro-resolving cytokine IL-10. ELISA analysis of media from these co-incubation experiments (Fig. 6a) confirmed that IL-10 expression was increased when macrophages were co-incubated with neutrophils and this was seen with both WT and CXCR2-deficient macrophages. Apoptotic neutrophils did not produce IL-10 when incubated alone (data not shown). These data confirm that CXCR2-deficient macrophages are fully capable of producing anti-inflammatory cytokines upon co-culture with apoptotic neutrophils and suggested that the reduced production of IL-10 at inflamed sites in CXCR2-deficient mice may be a consequence of impaired neutrophil recruitment and local interaction with macrophages.

To formally implicate impaired neutrophil recruitment in the phenotype that we have reported, we performed additional experiments in which we used the anti-Ly6G antibody to deplete circulating neutrophils in WT mice prior to intraperitoneal administration of zymosan. As shown in Fig. 6b,c, this antibody brought about essentially complete depletion of the neutrophilic (Ly6G+) population. However, and in contrast to the data obtained using CXCR2-deficient mice, neutrophil depletion in WT mice in fact impaired recruitment of macrophages to inflamed peritoneum. Together these data indicate that the basis for the phenotype observed appears to relate more to impaired recruitment of an alternative CXCR2+ve leukocyte population than to reduced uptake of apoptotic neutrophils by macrophages. We propose that this alternative CXCR2+ve population may comprise myeloid derived suppressor cells^29^. In this regard, it is notable that monocytic MDSC (M-MDSCs^30^) are categorised as being Ly6CHi^31^,^32^ and that a Ly6CHi myeloid cell population in WT peritoneum is missing in CXCR2-deficient peritoneum (see Fig. 5a, upper gate).

## Discussion

Given the ongoing attempts to target CXCR2 in inflammatory diseases and cancer^8^,^15^,^18^, it is of clear importance to fully understand and characterise the role of this receptor in the inflammatory response. Whilst our data re-iterate the importance of CXCR2 in supporting neutrophil recruitment to inflammatory sites, it also reveals a previously unanticipated role in controlling the magnitude of the macrophage-dependent inflammatory response. Specifically, our data show that cutaneous and peritoneal inflammation in CXCR2-deficient mice is characterised by a major reduction in the numbers of neutrophils present at the inflamed site and a corresponding increase in macrophage numbers. The speed with which macrophage numbers increase, especially in the zymosan model, suggests that this is not solely a consequence of increased local proliferation and is likely to be substantially dependent on de novo recruitment of these cells into the inflamed area This results in a transient exaggeration of the inflammatory response at both sites at time points after the peak of neutrophil recruitment^14^,^21^,^24^,^33^. Notably, in the skin, this is reflected in increased overall skin thickness which is accounted for entirely on the basis of increased dermal thickening. We did not observe epidermal thickening and interpret this as being a consequence of lack involvement of T cells in this response as it has previously been shown that T cells are important for inducing epidermal thickening during TPA-induced inflammation^21^,^34^.

We proposed two hypotheses to explain our observations. Firstly, in inflammation, neutrophil recruitment precedes macrophage recruitment^33^,^35^, and interaction of apoptotic neutrophils with subsequently recruited macrophages initiates resolution of the inflammatory response^36^. Accordingly the marked reduction of neutrophil recruitment in the CXCR2-deficient mice may contribute to the impaired induction of pro-resolution factors in recruited macrophages. However, experiments carried out using neutrophil-depleting antibodies suggested that the simple absence of neutrophil recruitment does not explain the phenotypes observed. Indeed, mice in which neutrophils were systemically depleted displayed reduced, and not exaggerated, macrophage recruitment. This has led us to conclude that the major role for CXCR2 in limiting inflammation relates to its expression on a non-neutrophil cell type. A potential candidate for such a cell is myeloid derived suppressor cells^29^ and their role in the phenotypes observed is further suggested by a specific reduction in a Ly6Chi monocytic cellular population^31^,^32^ along with the increased expression of pro-resolving cytokines such as TGFβ and IL-10^37^ in CXCR2-deficient mice.

Our data are in general agreement with previous studies demonstrating delayed wound healing in the CXCR2-deficient mice associated with reduced levels of TGF-β and enhanced monocyte recruitment at later time points^38^,^39^. However our data are in apparent contrast to a number of other studies demonstrating reduced acute inflammatory responses in CXCR2-deficient mice^40^. These are interpreted as being a consequence of altered neutrophil infiltration. However, these studies only looked at the earliest time points of the inflammatory response (typically 24 hours) and therefore missed the window of exaggerated inflammation identified in the present study. Specifically, our study mirrors that by Jamieson et al^14^ in demonstrating that neutrophil infiltration to the skin in response to a single TPA injection peaks after 24 hours, and that this is significantly reduced in CXCR2-deficient mice at all time points. However, in contrast to the data we describe here, the cellular content and overall morphology of the skin was not monitored later than 24 hours after the single TPA application (acute phase), due to the focus of the study. Importantly the study by Jamieson and colleagues was performed using CXCR2-deficient mice on the same C57Bl/6 background as used in the current study thus arguing against strain-specific contributions to the phenotype. Other studies have clearly demonstrated a beneficial effect of CXCR2-deficiency in mice during chronic models of inflammation^14^,^40^. However invariably these models involve continuous re-administration of inflammatory stimuli and thus are not immediately comparable with the current study that monitors later time points, after the peak of neutrophil recruitment, of the inflammatory response. Our experimental models may more closely recapitulate routine acute inflammatory responses where the body is challenged by a one-off stimulus resulting in a rapidly resolving inflammatory response.

In summary this manuscript reports previously unanticipated pro-inflammatory consequences of CXCR2-deletion in acute inflammatory responses. Our data therefore contribute to our overall understanding of the involvement of this receptor in the orchestration of the inflammatory response.

## Methods

### Mice and models of inflammation

Animal experiments were performed using female age-matched mice in accordance with the animal care and welfare protocols approved by the Animal Welfare and Ethical Review Board at the University of Glasgow and carried out under the auspices of a UK Home Office Project Licence. WT C57Bl/6 and CXCR2-deficient mice were bred in ‘specific pathogen free’ conditions in the animal facility of the Beatson Institute for Cancer Research. Animal numbers reflect the number required to provide robust statistical evidence for significant effects. To generate sterile cutaneous inflammation, mice were shaved in the lower dorsal region before vehicle (acetone) or 12-O tetradecanoyl phorbol 13-acetate (TPA) was applied to the shaved skin as described^21^,^34^. Mice were culled 24, 48 or 72 hours after application and the desired tissues dissected and analysed as described below. Peritoneal inflammation was generated by injection of 200 μl of 5 mg/ml zymosan (1 mg total) into the peritoneal cavity of each mouse, 48 hours later mice were culled and the peritoneal cellular contents analysed as described below.

### Skin thickness analysis, staining and histology

Shaved lower dorsal skin was pinched together and the thickness measured using calipers. Skin samples were harvested following TPA application, fixed in formalin, before being processed and embedded in paraffin wax. 5 μm sections were cut from each sample transferred to SuperFrost microscope slides (Thermo Scientific) and baked at 65 °C for 30 minutes. Sections were then de-waxed with xylene before re-hydration and staining with haematoxylin and eosin counterstain, de-hydration and mounting with DPX (Leica). Blinded measurement of dermal and epidermal thickness from skin sections was undertaken using Zen software (Zeiss).

### Inguinal lymph node analysis

Inguinal lymph nodes were removed, adhered onto SuperFrost microscopy slides (Thermo Scientific) and fixed overnight in formalin. Samples were then de-hydrated and incubated in xylene, before re-hydration and staining with Carmine solution, de-hydration and mounting with DPX. The lymph nodes were then imaged using bright-field microscopy on an EVOS microscope (Life Technologies) and the area occupied by the lymph nodes calculated from images using FIJI software^41^.

### Analysis of skin inflammatory cell infiltrate by immunocytochemistry

Skin was processed as above. Following re-hydration sections were washed in PBS, blocked with 3% H_2_O_2_ (SigmaAldrich) and 20% goat serum (SigmaAldrich), sections were then stained with anti MAC-2 antibody (CedarlaneLaboratories) or isotype control rat IgG_2a, κ_ (BD Biosciences) diluted 1/6000 in PBS containing 1% BSA (SigmaAldrich) and incubated overnight at 4 °C. Sections were washed before addition of goat anti-rat IgG secondary antibody (Vector Laboratories) (30 minutes, room temperature), further washing with PBS, addition of Extravidin Peroxidase (Sigma) diluted 1/200 in PBS containing 1% BSA (30 minutes, room temperature) before washing and signal development with DAB reagent (Vector laboratories) and quenching with tap water. Stained sections were then washed, counter-stained with haematoxylin, de-hydrated and mounted with DPX.

Alternatively, following de-waxing and re-hydration of sections to 95% ethanol, they were then stained with Astra Blue dissolved in 95% ethanol (1 hour, room temperature), before washing in ethanol containing 5% hydrochloric acid, counterstain in 1% Safranin-O (1 minute, room temperature), further washing, dehydration and mounting in DPX.

Ki67 and myeloperoxidase analysis was undertaken at the Diagnostic Services Unit, School of Veterinary Medicine at the University of Glasgow using tissues processed as above.

Blinded cell counts were undertaken using blinded bright-field microscopy undertaken at the indicated magnifications on a Zeiss Axiolimager M2 using Zeiss ZEN software.

### Skin protein content analysis

Skin samples from TPA experiments were ‘snap-frozen’ in liquid nitrogen, ground and re-suspended in PBS containing protease inhibitors (Life Technologies). Samples were then analysed using a mouse cytokine 20-plex kit (Life Technologies), used as directed in the kit and analysed with a Luminex 100 machine (Bio-Rad).

### Cytopsin analysis and Flow cytometry

Peritoneal cellular lavage, from mice injected with zymosan was spun and washed before re-suspension in FACS buffer (PBS + 0.1% BSA). Cells were then spun onto a microscopy slide using a cytocentrifuge before staining with 10% Giemsa solution and blinded cell morphology analysis. Alternatively, cell suspensions were stained with fixable viability dye (eBioscience) and antibodies against CD11b (eBioscience), F480 (eBioscience), Ly6C (Biolegend), CD206 (Biolegend) and SiglecF (BD Biosciences) (1/200 in FACS buffer). Cell suspensions were then washed in FACS buffer and run on a MACSQuant analyser (Miltenyi) and data processed using FlowJo software (Treestar Inc.).

### Macrophage-Neutrophil co-culture

1 ml of 3% Thioglycollate was injected into the peritoneum of mice and animals were culled either 18 hours (neutrophils) or 4 days (macrophages) later. For neutrophil purification the peritoneum was flushed with 5 ml of PBS containing 2 mM EDTA, cells were then separated and re-suspended in complete culture medium (DMEM with 10% FBS) in a tissue culture flask for 2 hours to remove adherent cells. Non-adherent cells (neutrophils) were removed and re-suspended to 5 × 10^6^ cells/ml and aged overnight. Preparations of neutrophils were routinely of >90% purity as assessed by FACS. Macrophages were isolated with the same approach: following incubation on a tissue culture flask non-adherent cells were removed to leave adherent macrophages (100,000/well). These were then washed and incubated with aged neutrophils (200,000/well) for either 12 or 24 hours. At each stage cell samples were taken for cytospin analysis to confirm cellular purification. Culture medium was then removed and stored at −80 °C before analysis undertaken using an Il-10 ELISA kit, as directed (Affymetrix, eBioscience).

## Additional Information

**How to cite this article**: Dyer, D. P. *et al*. CXCR2 deficient mice display macrophage-dependent exaggerated acute inflammatory responses. *Sci. Rep.* **7**, 42681; doi: 10.1038/srep42681 (2017).

**Publisher's note:** Springer Nature remains neutral with regard to jurisdictional claims in published maps and institutional affiliations.

## Supplementary Material

Supplementary Dataset 1
