# Corrigendum: CXCR2 deficient mice display macrophage-dependent exaggerated acute inflammatory responses

**DOI:** 10.1038/srep45423

**Published:** 2017-04-10

**Authors:** Douglas P. Dyer, Kenneth Pallas, Laura Medina-Ruiz, Fabian Schuette, Gillian J. Wilson, Gerard J. Graham

Scientific Reports 7: Article number: 42681; 10.1038/srep42681 published online: 02 16 2017; updated: 04 10 2017.

The original version of this Article contained a typographical error in the spelling of the author Laura Medina-Ruiz, which was incorrectly given as Laura Medina Ruiz. This has now been corrected in the PDF and HTML versions of the Article.

The Author Contributions statement now reads:

D.P.D., K.P., L.M-R., F.S. and G.W. performed experiments. G.J.G. conceived the study. G.J.G. and D.P.D. wrote the manuscript. All authors approved the manuscript prior to submission.

