# Supplementary material for: CXCR2 deficient mice display macrophage-dependent exaggerated acute inflammatory responses
Source: Sci Rep. 2017 Feb 16;7:42681. doi: 10.1038/srep42681 (PMC5311995; doi:10.1038/srep42681)
Supplement: Supplementary Dataset 1 [file srep42681-s1.doc]

**CXCR2 deficient mice display macrophage-dependent exaggerated acute inflammatory responses**

(Short title: Exaggerated inflammation in CXCR2-deficient mice)

Douglas P Dyer, Kenneth Pallas, Laura Medina Ruiz, Fabian Schuette, Gillian J Wilson, Gerard J Graham^*^.

**Supplementary Figure 1. CXCR2-deficient mice display increased skin cell proliferation and inguinal lymph node swelling during cutaneous inflammation.**

**(A)** TPA was applied to the dorsal skin of WT or CXCR2-deficient mice. **(i)** 24, **(ii)** 48 or **(iii)** 72 hours later, mice were culled and the skin harvested, fixed and analysed, by immunohistochemical staining, for the presence of ki67 (images shown at x40 magnification). **(B)** The number of Ki67-positive cells was counted from at least 5 fields of view per sample and plotted over time. **(C)** The inguinal lymph node was removed at each time point, fixed and stained and the area of the lymph node calculated. All experiments are plotted as the mean (±SEM) from groups containing 3-7 mice at different time points following TPA application and are representative of two independent experiments. **P*<0.05, ***P*<0.01 as determined using Students T test.

**Supplementary Figure 2.** **CXCR2-deficient mice display increased peritoneal inflammatory response.**

Zymosan was intra-peritoneally injected into mice, 48 hours later mice were culled and the peritoneum flushed with PBS. **(A)** Cells were then stained with antibodies to CD11b, the CD11b cells identified and then further gated for Ly6G, F480 and SiglecF expression using flow cytometry. **(B)** Quantitative data from the flow cytometric assessments. Data are plotted as the mean (±SEM) from groups containing at least 7 mice. These plots are representative of two independent experiments. **P*<0.05, ***P*<0.01, ****P*<0.001 as determined using Students T test.
